# CM-supplement network model for reducing the memory consumption during multilabel image annotation

**DOI:** 10.1371/journal.pone.0234014

**Published:** 2020-06-01

**Authors:** Jianfang Cao, Lichao Chen, Chenyan Wu, Zibang Zhang

**Affiliations:** 1 Department of Computer Science and Technology, Xinzhou Teachers University, Xinzhou, China; 2 School of Computer Science and Technology, Taiyuan University of Science and Technology, Taiyuan, China; University Tunku Abdul Rahman, MALAYSIA

## Abstract

With the rapid development of the Internet and the increasing popularity of mobile devices, the availability of digital image resources is increasing exponentially. How to rapidly and effectively retrieve and organize image information has been a hot issue that urgently must be solved. In the field of image retrieval, image auto-annotation remains a basic and challenging task. Targeting the drawbacks of the low accuracy rate and high memory resource consumption of current multilabel annotation methods, this study proposed a CM-supplement network model. This model combines the merits of cavity convolutions, Inception modules and a supplement network. The replacement of common convolutions with cavity convolutions enlarged the receptive field without increasing the number of parameters. The incorporation of Inception modules enables the model to extract image features at different scales with less memory consumption than before. The adoption of the supplement network enables the model to obtain the negative features of images. After 100 training iterations on the PASCAL VOC 2012 dataset, the proposed model achieved an overall annotation accuracy rate of 94.5%, which increased by 10.0 and 1.1 percentage points compared with the traditional convolution neural network (CNN) and double-channel CNN (DCCNN). After stabilization, this model achieved an accuracy of up to 96.4%. Moreover, the number of parameters in the DCCNN was more than 1.5 times that of the CM-supplement network. Without increasing the amount of memory resources consumed, the proposed CM-supplement network can achieve comparable or even better annotation effects than a DCCNN.

## Introduction

As multimedia technology rapidly develops and image acquisition devices become increasingly convenient, digital image resources have increased exponentially. How to rapidly retrieve objects of users’ interest from a large number of images has become an important research direction in the field of image processing. By automatically annotating the keywords reflecting semantic content, the auto-annotation technique narrows the gap between the low-level visual features of the image and the high-level semantic annotations [1]. It enhances the efficiency and accuracy of image retrieval and therefore gains broad application prospects in the fields of image and video retrieval, scene understanding and human-computer interaction [2]. Nevertheless, image auto-annotation remains a challenging topic due to the existence of “semantic gaps”, although it has long been a hot spot of research in the field of computer vision. As an image consists of complex semantic information, a single annotation often fails during the labeling task. Therefore, the adoption of a multilabel annotation approach becomes necessary. The labels in multilabel auto-annotation contain most of the information contained the annotated image. Thus, the image can be rapidly identified. In addition, this approach satisfies the retrieval requirement for images on the Internet with large-scale semantic information [3].

To date, scholars have proposed a number of models for image auto-annotation. These models can be roughly divided into three categories: generative models, nearest models and discriminative models. With generative models, the visual information of the image, such as colors, shapes, textures and spatial relations, is extracted. Then, the joint probability distribution of the visual features and tags or the conditional probability distribution of different tags is calculated, based on which the tags are scored for complete annotation [4]. The multiple Bernoulli relevance model and cross-media relevance model are typical examples of this category. [5, 6] Later, Moran et al. [7] proposed a modified sparse kernel learning continuous relevance model (SKL-CRM), which enhanced the performance of image annotation by learning the optimum combination among feature kernels. The fuzzy cross-media relevance model (FCRM) utilizes nonparametric Gaussian kernels to perform continuous estimation of the feature generation probability [8], which further improves the annotation accuracy. Although generative models involve a relatively simple annotation process, the gap between the low-level features and high-level semantics of the image, as well as semantic dependence, often leads to inaccurate joint probabilities [9]. In recent years, with the increase in training data, the nearest models have gained increasing popularity. In this category, image annotation is treated as a retrieval task, and the basic idea of the tag propagation mechanism is to find images that resemble the test image and then to annotate the test image with the tags corresponding to the resembling images [10]. The nearest models include the joint equal contribution (JEC) [11], tag propagation metric learning (TagProp_ML) [4] and two-pass K-nearest neighbor (2PKNN) [12] as typical examples. In particular, the 2PKNN Metric Learning (2PKNN_ML) method has been considered an advanced and representative method of nearest models in recent years. With the 2PKNN_ML method, after the semantic neighbor images of the test image are found, the distance weights between features are optimized via metric learning [13]. However, the nearest models suffer from the loss of much valuable information during image visual feature extraction, which is very likely to result in an unsatisfactory annotation effect [14]. Discriminative models treat each tag as a class and consider image annotation as a multiclassification task. Based on multiple classifier training, the models classify the test image into a class to which a certain tag belongs [15]. Typical examples of nearest models include the K-nearest neighbor (KNN) [16], K-means clustering [17], support vector machine (SVM) [18] algorithms, as well as the modified versions of these methods. However, all these methods are restricted and influenced by the number and training effect of the classifiers, particularly under the condition of imbalanced training samples, where the training effect of the classifiers corresponding to low-frequency tags is unsatisfactory, which in turn affects the total annotation accuracy; in addition, with the increase in the number of class tags, the number of required classifiers also increases, which makes the annotation model more complex [19].

With the continuous development of deep learning in recent years, convolutional neural networks (CNNs) have been extensively applied in the field of computer vision [20]. In 2012, Hinton et al used a multilayer CNN for image classification of the ImageNet dataset [21], and an impressive recognition effect was achieved [22]. Later, a large number of research projects focused on modifying CNNs in terms of structure and performance. For instance, GoogLeNet, developed by Google, was the champion in the 2014 large-scale image recognition competition [23], and in the ImageNet 1000 Challenge, the deep-level CNN-based computer vision system developed by the Visual Computing Group at Microsoft Research Asia outperformed humans in terms of object recognition and classification for the first time [24]. Furthermore, deep-level CNN-based discriminative models have also made certain achievements in multilabel image auto-annotation. Based on a CNN, Li et al. designed a softmax regression-based multilabel ranking loss function network, which greatly improved the auto-annotation effect compared with traditional methods [25]. Murthy et al. [26] proposed the linear regression-based CNN-regression (CNN-R) method, which optimized the model parameters via backpropagation (BP). Although these methods achieved improvement in image auto-annotation compared with traditional methods, most of them targeted improving the network itself or on single-label learning, seldom focusing on the application and improvement in multilabel learning-based image auto-annotation. Targeting the issue of low annotation accuracy of low-frequency tags caused by training sample imbalance in image semantic annotation, Cao et al. [27] designed a double-channel convolutional neural network (DCCNN) in 2019. However, due to the requirement of the simultaneous operation of the two channels during testing, great memory usage occurs.

Based on the aforementioned methods, the current study proposes a CM-supplement CNN, in which the characteristics of multilabel image annotation and the influence of training sample imbalance on image annotation were both taken into consideration. The proposed CNN possesses the following advantages: 1) The cavity convolution contained in the model expands the receptive field for feature extraction but without increasing the calculation burden and memory overhead; 2) Inception modules are incorporated into the model, which not only deepens the network but also increases the network width. These changes help extract features at different scales, strengthen the network robustness, and increase the network calculation speed along with a reduced memory overhead. 3) the supplement CNN-based structure can effectively solve the problem of the influence of the discarded negative information on the classification effect in traditional CNNs during image classification.

## Methodology

### Supplement CNN

**Fig 1:**
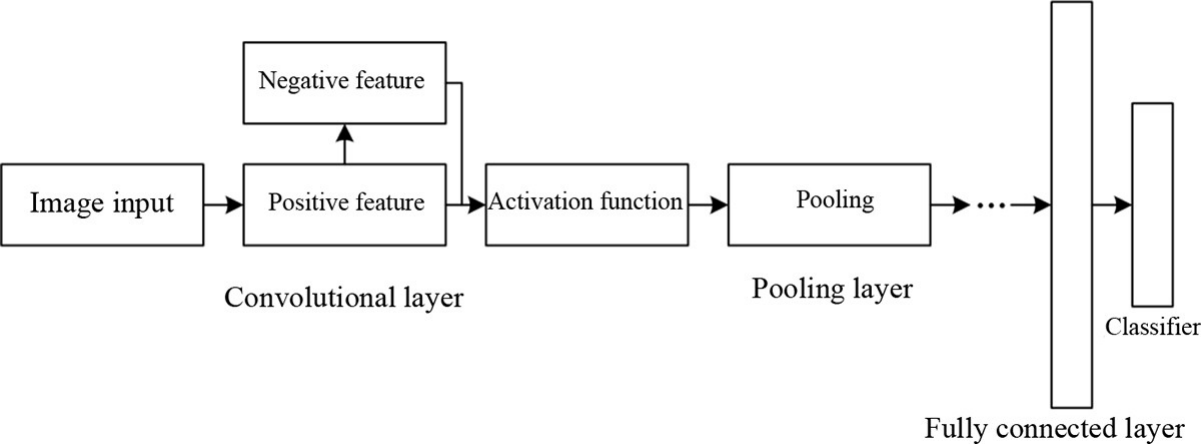
Structure of the supplement CNN.

The supplement CNN is a modified version of the traditional CNN [28]; its structure is shown in Fig 1.

As shown in Fig 1, the modifications of the current model mainly focus on the convolutional layer. The positive features extracted by the convolutional layer are reversed to obtain the negative features of the image, and then the positive and negative features are fused as input for the next layer. Compared with traditional CNNs, the current model maintains the negative information, which endows it with the ability to learn more features, thereby improving the image annotation and recognition accuracy.

Apart from the abovementioned difference, the supplement network also differs from the traditional CNN in the selection of the activation function. Normally, traditional CNN uses the ReLU function as the activation function. However, the ReLU function cannot maintain the negative information of the image, which can be observed from its expression. Instead, the supplement network uses ELU [29] as the activation function.

The ELU activation function (an exponential linear unit) is a correction function of ReLU. It keeps the negative information of the image by adding a nonzero output to a negative input, and its expression is as follows:

$$f(x)=\{\begin{array}{l} \partial (e^{x}-1)\;x\leq 0\\ \;x\;x>0 \end{array}$$ (1)

where *x* represents the feature map of the image.

According to Eq (1), the ELU function contains a negative exponential term, which can prevent the appearance of silent neurons, thereby enhancing the overall learning efficiency of the network.

### CM-supplement CNN

#### Network structure design

**Fig 2:**
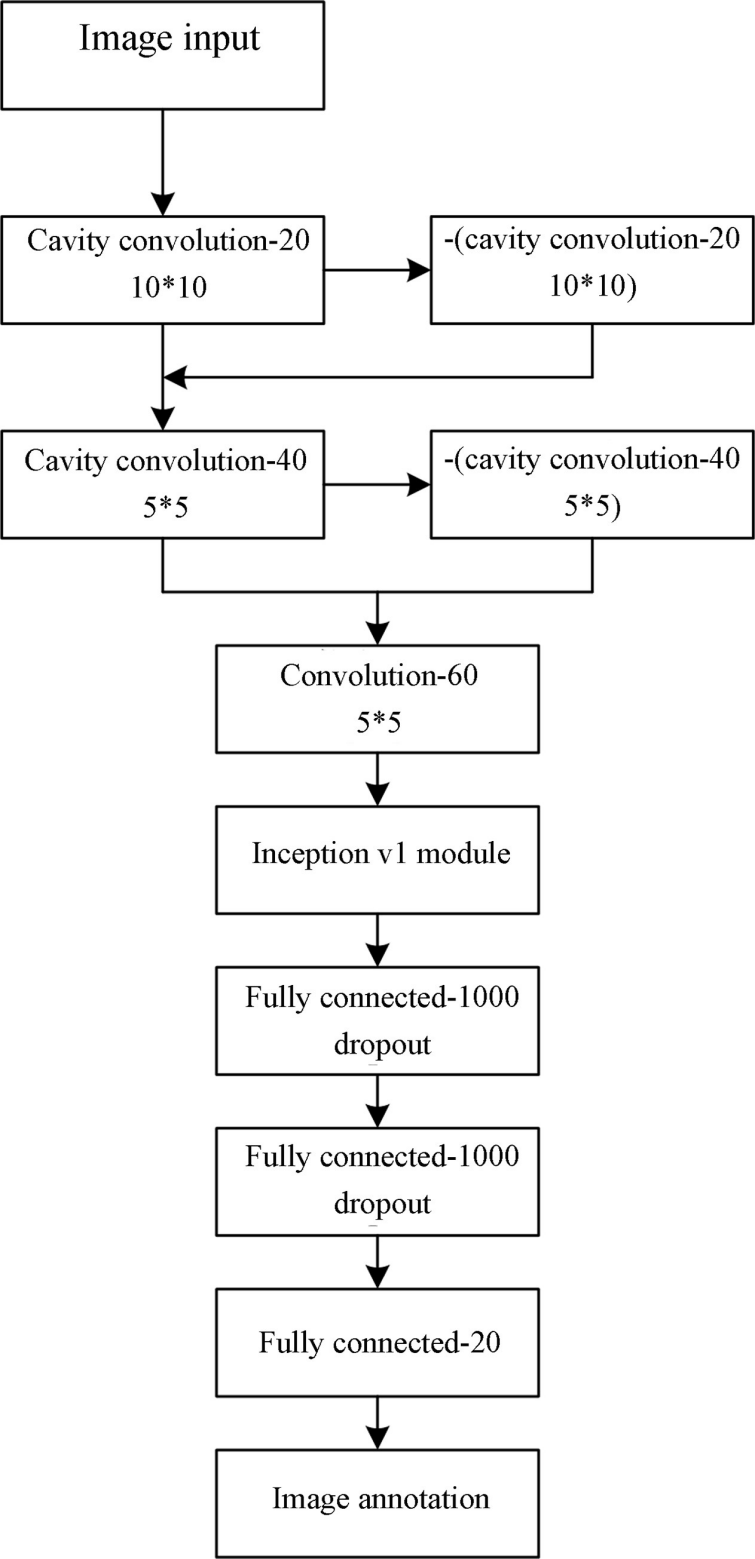
The CM-supplement model.

The structure of the proposed CM-supplement network in this study is shown in Fig 2.

The proposed model is a seven-layer structure with four convolutional layers and three fully connected layers. The detailed information is as follows.

In layers 1 and 2, cavity convolutions with an expansion coefficient of 2 are used as the convolution kernels. This treatment can expand the receptive field, without increasing the network memory overhead and calculation burden, to extract more image features. For example, when the expansion coefficient of a convolutional kernel with a size of 3*3 is set to 2, its receptive field will change from 3*3 to 7*7, but without increasing the number of the parameters. Furthermore, pooling layers do not need to be set for a network containing cavity convolutions because the function of pooling layers is just to expand the receptive field for feature extraction. In addition, some feature information can be lost during receptive field expansion by pooling layers, while this drawback can be well remedied by cavity convolutions.

In addition, in both layer 1 and layer 2, a supplement model structure is used to obtain the negative features of the image for their transfer into the next layer. The implementation of the supplement model is simple and does not need extra parameters to be added (and only involves the reversal operation of the features extracted by the cavity convolution followed by the fusion between the reversed features and the original ones). Therefore, the supplement model was used as a component of the CM-supplement model in this study.

In layer 3, a common CNN is used for the transition from the fusion structure of the cavity convolution with the supplement structure to the Inception module. Furthermore, its use can reduce the calculation burden by reducing the dimensions before the features enter the Inception module.

The fourth layer contains Inception modules. The reason for the incorporation of the Inception module is that it possesses the capability to extract image features at different scales. Furthermore, the number of parameters and computational load of the module are fewer than those of commonly used CNN convolutions. In this study, the dataset for training was PASCAL VOC 2012. Considering that the amount of training data was not large, the Inception v1 module was utilized for its simple structure with a small number of parameters.

Fully connected layers constitute layers 5–7. According to reference [27], a dropout layer was added after layers 5 and 6 to prevent overfitting.

#### Principles of the modified algorithm

Image annotation consists of two key steps: image feature extraction and the establishment of the mapping relation between the features and tags. In essence, the CM-supplement network designed in this study is subject to modifications within the scope of feature extraction: The cavity convolution, Inception v1 structure and supplement structure are combined for the extraction of finer multiscale features to enhance the accuracy of image annotation. These modifications are embodied in the forward propagation process of the image input model during network training as well as in the network parameter adjustment process according to the difference between the network-predicted outcome and the actual image tag, i.e., the backpropagation process.

(1) Forward propagation process

The forward propagation of the CNN model is primarily accomplished by the operation of the convolutional layer and pooling layer. Suppose that the first layer is a convolutional layer; its calculation formula is as follows:

$$x_{j}^{l}=f\left(\sum_{i\in M_{j}}x_{i}^{l-1}*k_{ij}^{l}+b_{j}^{l}\right)$$ (2)

where *M*_*j*_ represents the input image dataset, $x_{j}^{l}$ represents the *j*th feature map of the *i*th layer, * denotes the convolution operation, $x_{i}^{l-1}$ is the *i*th feature map of the (*l-1*)st layer, $k_{ij}^{l}$ represents the convolution kernel that connects the *j*th feature of the *l*th layer with the *i*th feature of the (*l-1*)st layer, $b_{j}^{l}$ represents the bias, and *f*(⋅)represents the nonlinear activation function of the neuron.

Compared with the forward propagation process of the traditional CNN structure, the adoption of the supplement structure enables the CM-supplement network to fuse the positive and negative features of the image, and the calculation formula is as follows:

$$x_{j}^{l}=f(\sum_{i\in M_{j}}x_{i}^{l-1}*k_{ij}^{l}+b_{j}^{l})+f(-(\sum_{i\in M_{j}}x_{i}^{l-1}*k_{ij}^{l}+b_{j}^{l}))$$ (3)

where *M*_*j*_ is the set of the feature maps output by the preceding layer, $x_{j}^{l}$ is the *j*th feature map of the *l*th layer calculated based on the (*l-1*)st layer, * denotes the convolution operation, $k_{ij}^{l}$ is the convolutional kernel corresponded by the *j*th feature map of the *l*th layer based on the convolution calculation of the *i*th feature map of the (*l-1*)st layer, $b_{j}^{l}$ represents neural bias, and *f*(⋅) is the activation function that maps the data onto a certain range after the convolution operation. In Eq (3), the plus symbol does not mean a simple mathematical operation but a connection (concatenation) operation.

(2) Backpropagation process

The errors in CNN backpropagation primarily consist of output layer errors and hidden layer errors. Suppose that the target function of CNN is a variance cost function as follows:

$$J(w,b,x,y)=\frac{1}{2}{\left\|y-h_{w,b}(x)\right\|}^{2}$$ (4)

where *w* represents the weight, *b* represents the bias, *x* is the input feature of the image, *y* represents the output value and *h*_*w*,*b*_(*x*) represents the actual value. The specific error adjustment process is described as follows:

- Calculation formula for the output errors:
  $$\delta_{j}^{l}=\frac{\partial J}{\partial z_{j}^{l}}$$ (5)
  where $\delta_{j}^{l}$ is the error produced by the *j*th neuron of the *l*th layer and $z_{j}^{l}$ represents the input of the *j*th neuron of the *l*th layer, which is associated with the weight and bias, respectively.
- Calculation formula for error propagation from the pooling layer to the convolutional layer:
  $$\delta_{j}^{l}=\beta_{j}^{l}(upsample(\delta_{j}^{l+1})\circ f'(x_{j}^{l}))$$ (6)
  where $\beta_{j}^{l}$ is the feature of the *j*th neuron of the *l*th layer, $\delta_{j}^{l}$ is the error of the *j*th neuron of the *l*th layer, *f*'(⋅) is the derivative of the activation function, *unsample*(⋅)denotes the upsampling operation, and ∘ represents the multiplication operation.
- Calculation formula for error updating from the convolutional layer to the pooling layer:
  $$\delta^{l}=\delta^{l+1}*rot180(W^{l})\circ f'(z^{l})$$ (7)
  where *f*'(⋅) is the derivative of the activation function, *rot*180(⋅) means 180-degree rotation of the convolution kernel, and *δ*^*l*+1^ represents the error in the next layer.

Then, the updated equations of *W* (weight) and *b* (bias) of the network are as follows:

$$W_{new}^{l}=W_{old}^{l}-\alpha *\frac{\partial J}{\partial W^{l}}=W_{old}^{l}-\alpha *(a^{l-1}*\delta^{l})$$ (8)

$$b_{new}^{l}=b_{old}^{l}-\alpha *\frac{\partial J}{\partial b^{l}}=b_{old}^{l}-\alpha *(\sum_{u,v}{\left(\delta^{l}\right)}_{u,v})$$ (9)

where $W_{new}^{l}$ is the updated weight, $W_{old}^{l}$ is the weight before updating, *α* is the learning rate of the network, $b_{new}^{l}$ is the updated bias, and $b_{old}^{l}$ is the bias before updating.

As the proposed CM-supplement network extracts more feature information during the forward propagation process than during the backpropagation process, predicted outcomes close to actual values can be obtained, and the error between the predicted value and the actual value can then be reduced, which in turn influences the backpropagation process and accelerates the convergence rate of the network.

### Multilabel image annotation

**Fig 3:**
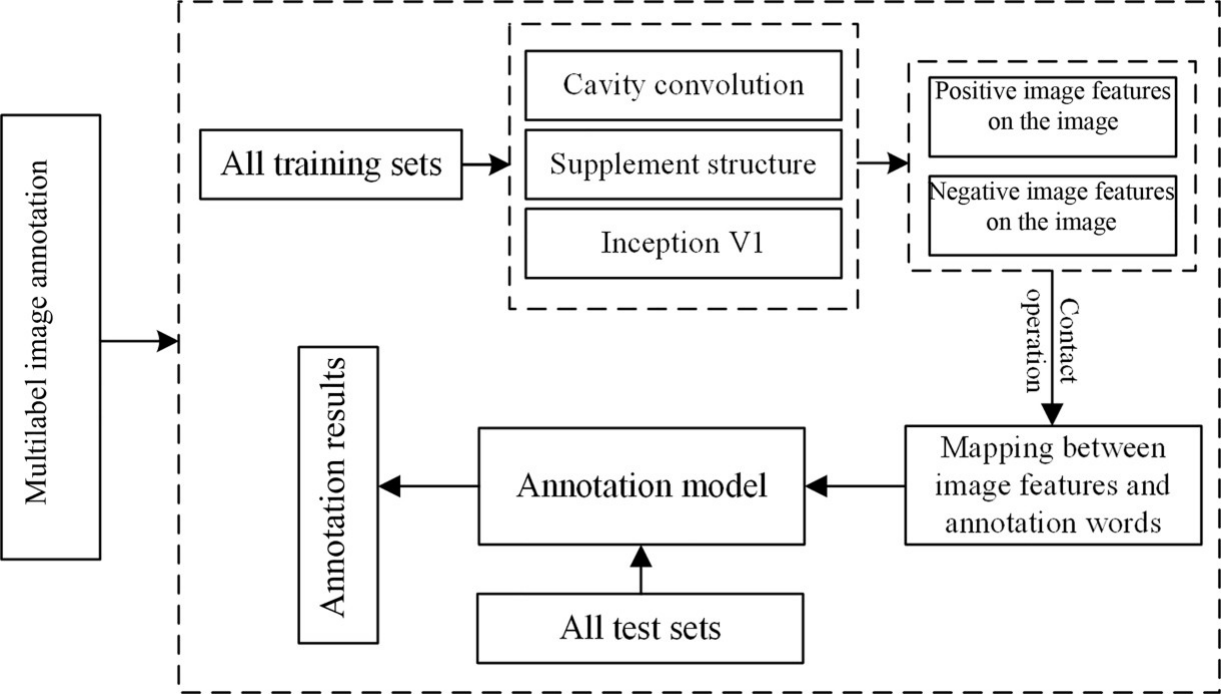
Schematic diagram of the image annotation part of the CM-supplement network.

The framework of automatic multilabel annotation proposed in this study is shown in Fig 3. First, the positive and negative features of all the training sets are extracted with the proposed CM-supplement network. Second, the extracted features are concatenated to establish the semantic mapping between the image features and the tags, and the mapping is used as the input to construct the annotation model. Then, the nonannotated images are input into the trained network for image tag predictions.

The proposed framework modifies the convolutional layer of the CNN model: The feature map obtained by the convolutional layer is negated, and the negative feature map combined with the original feature map is introduced into the ELU activation function and then transferred to the next layer. Such treatment enables the framework to obtain more feature information of the image than other methods and reduces the total error, which strengthens the learning of valuable feature information and makes the acquisition of the semantic information implied in the image easier, thereby benefiting automatic image annotation.

The algorithm is described as follows:

Step 1: Count the number of samples corresponding to each tagging word in the training set and determine the tag set;

Step 2: Employ the convolutional layer and pooling layer for forward propagation and combine the cavity convolution operation, the Inception v1 structure and the supplement structure to extract the positive and negative features;

Step 3: Concatenate the positive and negative features of the image;

Step 4: Realize backpropagation by updating the errors of the output layer and hidden layer and continuously adjust the network parameters according to the differences between the network-predicted results and the actual tags of the image;

Step 5: Repeat Steps 2–4, and train the network model until it is stable;

Step 6: Input the image to be tested into the trained network for annotation to obtain the annotation outcomes.

## Experiments and result analysis

### Experimental environment and design

The experimental PC environment consisted of a Windows 10 system, i7-8750 processors, 8 GB of memory, and an NVIDIA GTX1060 GPU. The Python programming language combined with the TensorFlow deep learning framework from Google was adopted to realize the idea behind the algorithm proposed in this study. The proposed CNN model was established.

For comparison, the parameters of the CM-supplement model were basically consistent with those of the CNN in reference [27]. In layer 1, the size of the convolution kernels (n = 20) was set to 10*10 with a step length of 4. In layer 2, the step length of the convolution kernels was set to 2 with a size of 5*5 (n = 40). In layer 3, 60 common convolution kernels were used with sizes and step lengths and 5*5 and 1, respectively. Following the third convolutional layer was a pooling layer with a size of 7*7 and a step length of 3. The Inception v1 structure followed the pooling layer. The last three layers were fully connected layers. A dropout layer was added following the first two fully connected layers to prevent overfitting, whose parameter set used the k-fold cross-validation method. After repeated experiments, k was determined to be 10 in this study. The learning rate of the CM-supplement model was set to 0.00001, which was two orders of magnitude lower than that of the compared CNN model. The reason is that when the learning rate of the CM-supplement network is set to 0.001 during training, the NaN error will occur in the corresponding loss function, which means that the obtained loss value would go beyond the range the computer can display, thereby becoming the non-numeric type. By reducing the learning rate, the loss function values can return to normal.

The mean average precision (MAP) was used as the assessment index for multilabel image annotation [27], and its value is the average of the average precision values.

$$AP=\frac{1}{11}\sum_{r\in \{0,0.1,\cdots ,1\}}Pinterp(r)$$ (10)

where *r* represents a group of set thresholds, *AP* is the average accuracy rate, and *Pinterp*(*r*) represents the maximal precision value corresponding to each threshold:

$$Pinterp(r)=max_{\tilde{r}:\tilde{r}\geq r}p(\tilde{r})$$ (11)

where $\tilde{r}$ denotes a certain threshold and $p(\tilde{r})$ is the accuracy rate corresponding to each $\tilde{r}$.

$$MAP=\frac{\sum_{q=1}^{Q}AP(q)}{Q}$$ (12)

where *q* represents a certain category and *Q* is the number of categories.

The adoption of the MAP as an assessment index was based on the consideration that it can overcome the limitation of the isolated accuracy rate, recall rate and F1-score to obtain an index that reflects the overall performance.

### Experimental data source

To validate the effectiveness of the proposed CM-supplement network model, the free, open access PASCAL VOC dataset [30] (http://host.robots.ox.ac.uk/pascal/VOC/voc2012/index.html) was used for the experiment. PASCAL VOC 2012 contains a total of 20 categories and 22,531 images, with approximately 1,000 images in each category.

### Results analysis

In this study, comparisons were made in terms of the annotation accuracy rate and memory consumption.

#### Annotation accuracy

(1) Comparison after 100 training sessions

The accuracy rates of the CM-supplement network, traditional CNN, DCCNN [27], the methods from references [29], [31], [32] and [33] after 100 iterations of training are summarized in Table 1.

**Table 1:** Comparisons of the annotation accuracy rates for each category of the PASCAL VOC 2012 dataset based on different algorithms after 100 training iterations.

Image category	Annotation accuracy rate						
	CNN	DCCNN [27]	Method from reference [31]	Method from reference [29]	Method from referenc [32]	Method from reference [33]	Method proposed in this study
plane	0.983	0.999	0.919	0.985	0.993	0.981	1.0
bike	0.877	0.973	0.448	0.902	0.973	0.452	0.978
bird	0.918	0.984	0.912	0.925	0.981	0.911	0.991
boat	0.920	0.972	0.639	0.919	0.971	0.647	0.976
bottle	0.722	0.892	0.750	0.822	0.889	0.720	0.892
bus	0.920	0.980	0.928	0.925	0.978	0.919	0.985
car	0.819	0.939	0.874	0.832	0.939	0.873	0.943
cat	0.916	0.970	0.921	0.934	0.965	0.922	0.978
chair	0.668	0.804	0.345	0.708	0.803	0.408	0.823
cow	0.999	1.0	0.851	0.999	0.999	0.862	1.0
dining table	0.570	0.757	0.693	0.695	0.748	0.627	0.803
dog	0.894	0.971	0.871	0.898	0.976	0.883	0.992
horse	0.927	0.978	0.886	0.938	0.969	0.876	0.990
motorbike	0.849	0.931	0.743	0.866	0.942	0.746	0.977
person	0.871	0.957	0.772	0.894	0.956	0.791	0.969
potted plant	0.729	0.881	0.654	0.805	0.878	0.639	0.880
sheep	0.960	0.993	0.851	0.963	0.991	0.849	0.991
sofa	0.618	0.827	0.332	0.666	0.824	0.307	0.828
train	1.0	0.999	0.813	1.0	1.0	0.825	1.0
tv monitor	0.746	0.866	0.721	0.765	0.867	0.738	0.896
MAP value	0.845	0.934	0.746	0.872	0.932	0.749	0.945

As shown in Table 1, after 100 training cycles, the proposed CM-supplement model achieved the highest MAP (94.5%), which was an increase of 10.0, 1.1, 19.9, 7.3, 1.3 and 19.6 percentage points compared with the traditional CNN, DCCNN and the methods from references [29], [31], [32], [33], respectively. Due to feature extraction based on artificial selection, the methods used in references [31] and [33] achieved a low accuracy rate when encountering some complex categories, such as the bike, chair, dining table, potted plant and sofa images. In contrast, the convolution neural network-based methods, such as the CNN and the methods used in references [27], [29] and [32], noticeably improved the accuracy rate, as the extracted image features by these methods are more abstract and more comprehensive than those features extracted by the other methods, which are closer to the high-level semantics of the images understood by human beings. The proposed CM-supplement network achieved an even higher accuracy rate because it can extract more feature information during forward propagation than these methods; therefore, the predicted results are closer to the actual values.

(2) Comparison of the changes in the MAP according to the number of iterations

**Fig 4:**
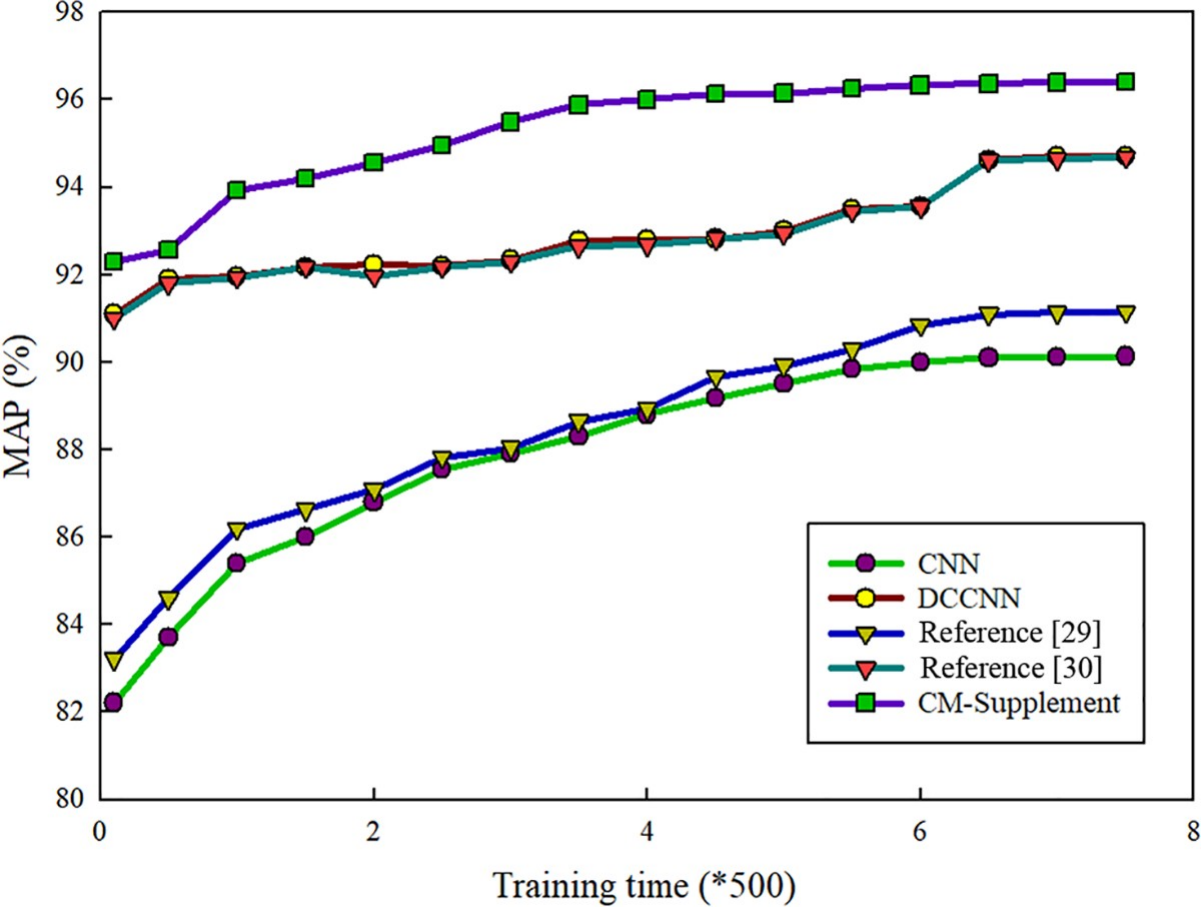
Comparisons of the MAPs of the CM-supplement model and other methods based on convolution neural networks (CNN, DCCNN and references [29] and [32]). The horizontal axis represents the number of iterations implemented during network training, and the vertical axis represents the increase in the number of iterations. MAP, mean average precision.

Because the methods used in references [27] and [33] are based on artificial feature extraction, when they encounter a large number of samples, the training time will be long. Therefore, the changes in the MAP according to number of iterations were compared only between the proposed CM-supplement network and the other convolution neural network methods, and the results are shown in Fig 4.

As shown in Fig 4, the CM-supplement network converged much faster than the other four methods, especially the methods of CNN and literature [29]. Even at the beginning of the training, the CM-supplement model obtained a high accuracy rate, which showed a relatively stable tendency. Furthermore, after training and stabilization, the final MAP of the CM-supplement network remained higher than that of any other model.

(3) Comparisons of the annotation accuracy rates after stabilization training

**Fig 5:**
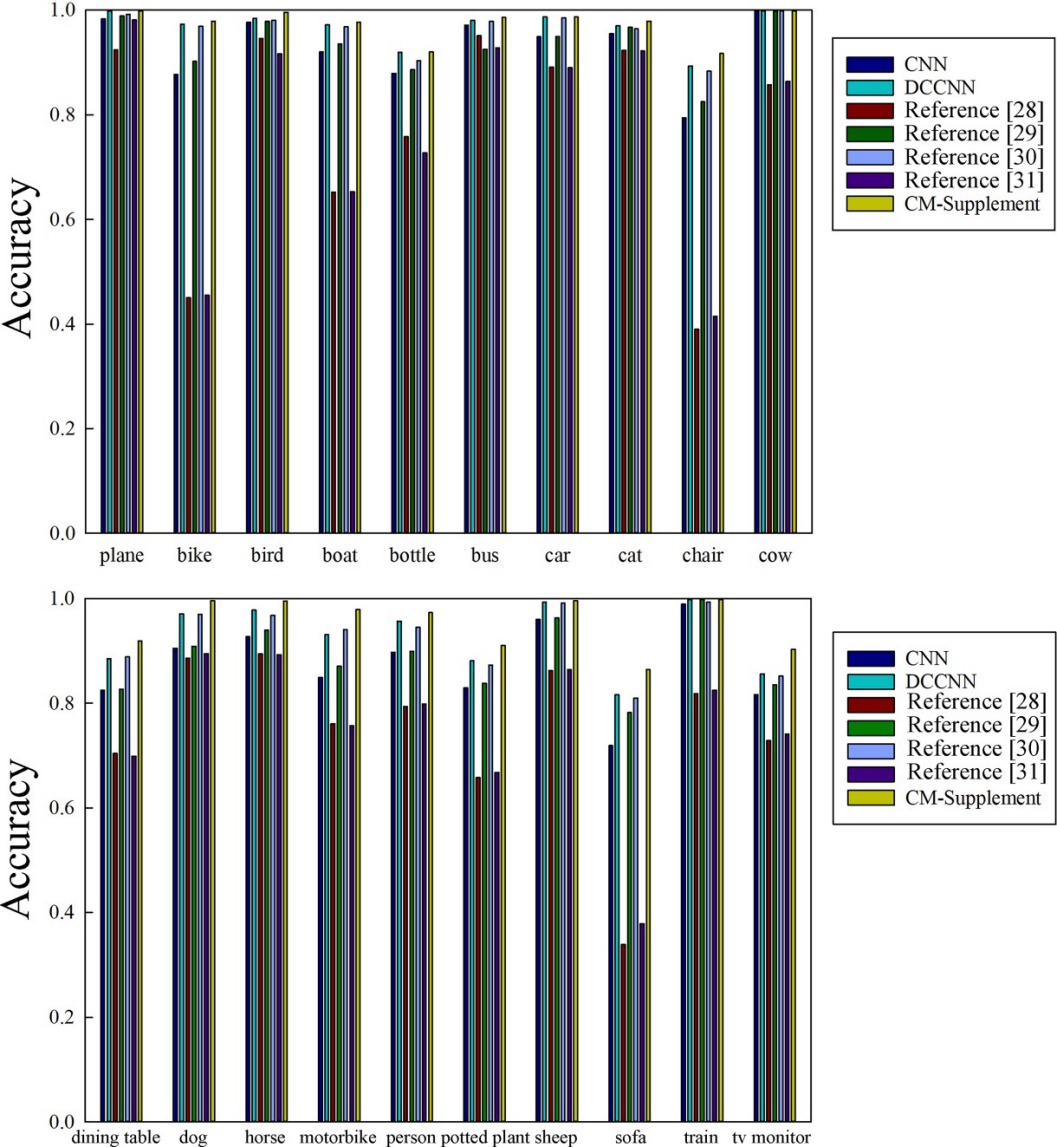
Comparisons of the annotation accuracy rates of the three networks for PASCAL VOC 2012 dataset after stabilization.

To further validate the performance of the proposed model, the poststabilization annotation accuracy rates of the seven models based on convolutional neural networks for the 20 categories in the PASCAL VOC 2012 dataset were compared (Table 2 and Fig 5).

**Table 2:** Comparisons of the annotation accuracy rates for each category in the PASCAL VOC 2012 dataset after stabilization training.

Image category	Annotation accuracy rate						
	CNN	DCCNN [27]	Method from reference [31]	Method from reference [29]	Method from reference [32]	Method from reference [33]	Method in this study
plane	0.983	0.999	0.924	0.989	0.992	0.981	1.0
bike	0.877	0.973	0.451	0.902	0.969	0.455	0.979
bird	0.977	0.984	0.946	0.978	0.980	0.916	0.996
boat	0.920	0.972	0.652	0.935	0.968	0.653	0.977
bottle	0.879	0.919	0.758	0.886	0.903	0.727	0.920
bus	0.971	0.980	0.951	0.925	0.978	0.928	0.986
car	0.949	0.987	0.891	0.949	0.985	0.890	0.987
cat	0.955	0.970	0.923	0.967	0.964	0.922	0.979
chair	0.794	0.893	0.39	0.825	0.883	0.415	0.917
cow	0.999	1.0	0.857	0.999	0.999	0.864	1.0
dining table	0.825	0.885	0.704	0.827	0.889	0.699	0.919
dog	0.905	0.971	0.886	0.909	0.970	0.895	0.996
horse	0.927	0.978	0.894	0.940	0.968	0.893	0.995
motorbike	0.849	0.931	0.761	0.871	0.941	0.757	0.979
person	0.897	0.957	0.794	0.899	0.945	0.799	0.973
potted plant	0.829	0.881	0.658	0.838	0.873	0.668	0.910
sheep	0.960	0.993	0.862	0.963	0.991	0.864	0.996
sofa	0.719	0.816	0.339	0.782	0.810	0.379	0.864
train	0.989	1.0	0.818	1.0	0.993	0.825	1.0
tv monitor	0.816	0.856	0.729	0.835	0.852	0.741	0.903
MAP value	0.901	0.947	0.759	0.911	0.943	0.764	0.964

As shown in Table 2, the accuracy rates of the methods from references [31] and [33] were noticeably lower than those involving convolution neural network-based methods. For the labels with sufficient training and a high original annotation accuracy rate, such as plane, cat, cow and train, the annotation accuracy rates of the five networks were comparable. However, for those labels corresponding to a relatively small number of training images, the CM-supplement network, the DCCNN and the methods from references [29] and [32] showed a noticeably better annotation effect compared with the CNN, with the best performance was observed for the CM-supplement model. For instance, compared with the traditional CNN, the CM-supplement network increased the annotation accuracy rate for ‘chair’ by 12.3 percentage points, ‘dining table’ by 9.4 percentage points, ‘motorbike’ by 13 percentage points, ‘potted plant’ by 8.1 percentage points, ‘sofa’ by 14.5 percentage points, and ‘TV monitor’ by 8.7 percentage points.

As shown in Fig 5, the annotation rates of the CM-supplement network for the 20 annotated words were much higher than those of the methods from reference [31] and [33], were superior to those of the methods from reference [29] and [32] and were comparable to those of the DCCNN. Overall, the average accuracy rate of the former was only 1.7 percentage points higher than that of the latter. However, the proposed model had a smaller number of parameters and consumed fewer memory resources than the DCCNN.

#### Comparisons of the amount of memory consumed

Although the CM-supplement and DCCNN models were comparable in terms of the annotation effect, they differed greatly in the number of network parameters (Tables 3 and 4).

**Table 3:** The number of parameters in each layer of the DCCNN.

DCCNN	Parameters corresponding to each layer of the channel		Number of parameters
256*256 input image	Channel 1	Channel 2	
Convolution layer 1	Convolution kernel [10, 10, 3, 20]	Convolution kernel [12, 12, 3, 20]	((10*10*3+1)+(12*12*3+1))*20 = 14680
Convolution layer 2	Convolution kernel [5, 5, 20, 40]	Convolution kernel [5, 5, 20, 40]	((5*5+1)+(5*5+1))*40 = 2080
Convolution layer 3	Convolution kernel [6, 6, 40, 60]	Convolution kernel [5, 5, 40, 60]	((6*6+1)+(5*5+1))*60 = 3780
Fully connected layer 1	[6*6*60, 1000]	[5*5*60, 1000]	(6*6*60+5*5*60)*1000+2000 = 3662000
Fully connected layer 2	[1000, 1000]	[1000, 1000]	1000*1000*2+2000 = 2002000
Output	[1000, 20]	[1000, 20]	1000*20*2 = 4000
Total number of parameters	14680+2080+3780+3662000+2002000+4000 = 5688540		

**Table 4:** The number of parameters of each layer of the CM-supplement network.

CM-supplement network	Parameters in each layer	Number of parameters
256*256 input image		
Cavity convolution layer 1	Convolution kernel [10, 10, 3, 20]	(10*10*3+1)*20 = 6020
Cavity convolution layer 2	Convolution kernel [5, 5, 20, 40]	(5*5+1)*40 = 1040
Cavity convolution layer 3	Convolution kernel [5, 5, 40, 60]	(5*5+1)*60 = 1560
Inception v1	(1*1+1)*4+3*3+5*5	(1*1+1)*4+3*3+5*5+2 = 44
Fully connected layer 1	[10*1*256, 1000]	10*1*256*1000+1000 = 2561000
Fully connected layer 2	[1000, 1000]	1000*1000+1000 = 1001000
Output	[1000, 20]	1000*20 = 20000
Total parameter number	6020+1040+1560+44+2561000+1001000+2000 = 3572664	

For the parameters and the number of parameters:

(1) Convolutional layer: Convolution kernel [height, c_height; width, c_width; number of input channels, channel_input; number of output channels, channel_output]

Number of parameters in the convolutional layer = (c_height*c_width*channel_input+1)* channel_output

(2) Number of parameters in the Inception v1 layer = number of feature maps *the size of the convolution kernel

(3) Fully connected layer: [number of input feature maps, fc_inputfeature; number of output feature maps, fc_outputfeature]

Number of parameters in the fully connected layer = fc_inputfeature*fc_outputfeature+fc_outputfeature

(4) Output layer: [number of the input feature maps, out_ inputfeature; number of image categories, cata_num]

Number of parameters in the output layer = out_ inputfeature*cata_num

As shown in Tables 3 and 4, the total number of parameters in the DCCNN was more than 1.5 times that of the CM-supplement network. This result fully indicates the effectiveness of the proposed model in image auto-annotation; it achieved a comparable or even better annotation effect compared with the DCCNN but without increasing the amount of memory resources consumed.

### Comparison with the actual annotation effects

To validate the annotation effect of the method proposed in this study, the automatic annotation outcomes of the proposed CM-supplement network for high-frequency and low-frequency words were compared with those based on the methods from reference [27] and [32], as the latter two methods achieved a relatively high annotation accuracy rate. The results are summarized in Tables 5 and 6.

**Table 5:** Comparisons of the annotation effects from different algorithms for high-frequency words.

Image example	Auto-annotation outcomes		
	DCCNN [27]	Reference [32]	Method proposed in this study
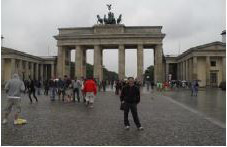	people	people	people, horse
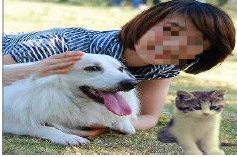	dog, people, cat	dog, people	dog, people, cat
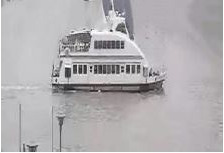	boat	boat	boat, people
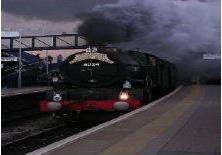	train	train, people	train, people
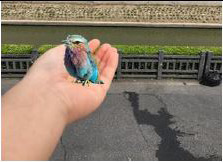	bird	bird, people	bird, people
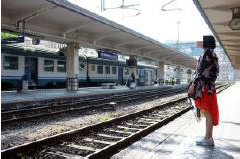	people, train	people	people, train
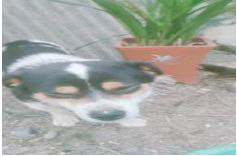	dog, potted plant	dog, potted plant	dog, potted plant
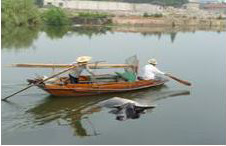	boat, people	boat, people	boat, cow, people
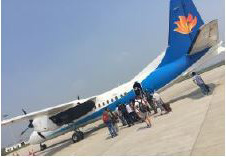	plane, people	plane, people	plane, people
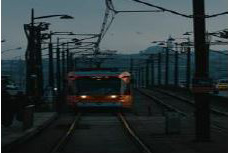	train	train	train, bus

All images in this table are sourced from the photos taken by the current team. For copyright consideration, they are similar but not identical to the original images sourced from PASCAL VOC 2012 and are therefore for illustrative purposes only.

**Table 6:** Comparisons of the annotation effects from different algorithms for low-frequency words.

Image example	Auto-annotation outcomes		
	DCCNN[27]	Reference [32]	Method proposed in this study
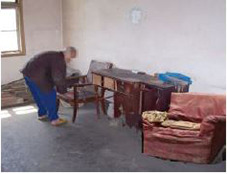	people	people	people, sofa, chair
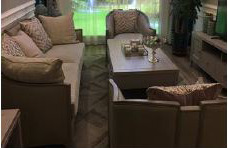	sofa	sofa	sofa, potted plant, chair, tv monitor
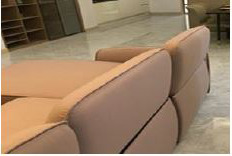	chair, sofa	sofa	chair, sofa, dining table
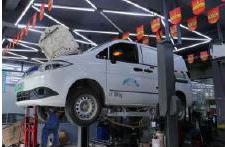	people, car	people, car	people, car, tv monitor
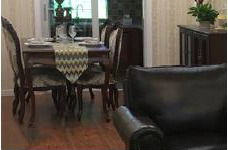	chair, dining table	chair, dining table	potted plant, chair, dining table, sofa
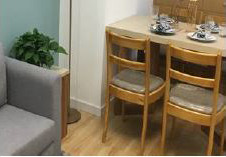	dining table, chair	dining table	dining table, chair, potted plant, sofa
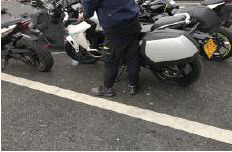	motorbike	motorbike	people, motorbike, chair
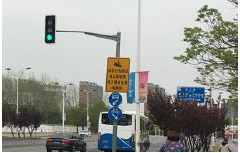	people, bus	people, bus	people, bus, car, bike
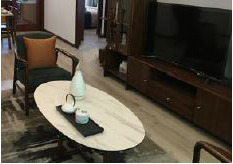	chair, tv monitor	chair, tv monitor	sofa, chair, tv monitor, dining table
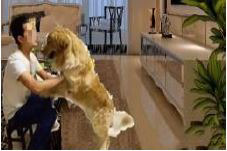	chair, people	chair, people,	dog, chair, people, sofa, potted plant

All images in this table are sourced from the photos taken by the current team. For copyright consideration, they are similar but not identical to the original images sourced from PASCAL VOC 2012 and are therefore for illustrative purposes only.

Table 5 shows that the annotation outcomes of the three methods were similar in terms of high-frequency words. However, as shown in Table 6, compared with the other two methods, the CM-supplement network exhibited better image descriptions and more complete annotations than the other methods. Moreover, for low-frequency words, such as chair, sofa, tv monitor and dining table, the proposed method had a higher recognition rate. Therefore, although the DCCNN and the method from reference [32] also achieved a more accurate and comprehensive description of the images, the proposed method provided the most complete description of the images.

## Conclusion

In this study, a CM-supplement network was proposed for multilabel image auto-annotation based on the characteristics of multilabel learning as well as the consideration of the memory resource consumption, and the typical, frequently used multilabel image dataset PASCAL VOC 2012 was selected for result validation. The comparisons between the proposed CM-supplement network and double-channel CNN proved the improved overall annotation efficiency and reduced memory resource consumption of the model proposed in this study.

Based on the outcomes of this study, future research may be carried out in the following three aspects:

(1) Larger-scale datasets can be used. As CNN training requires a large amount of data, larger datasets can leverage the advantages of neural networks. They aid in obtaining better parameters and avoiding overfitting, which can further improve the stability of the solution;

(2) Due attention can be given to word-word symbiotic relations and the image-image distance to further enhance the annotation accuracy rate;

(3) A semi or even unsupervised strategy can be adopted. As the resources of labeled images are limited in quantity, artificial annotation requires a large amount of labor and materials resources. The introduction of a semi- or unsupervised strategy can realize satisfactory annotation outcomes by resorting to a small portion of labeled images.
